# Anti-Inflammatory Therapy for Atherosclerosis: Focusing on Cytokines

**DOI:** 10.3390/ijms22137061

**Published:** 2021-06-30

**Authors:** Anastasia V. Poznyak, Dwaipayan Bharadwaj, Gauri Prasad, Andrey V. Grechko, Margarita A. Sazonova, Alexander N. Orekhov

**Affiliations:** 1 Institute for Atherosclerosis Research, Skolkovo Innovative Center, 121609 Moscow, Russia; 2 Academy of Scientific and Innovative Research, CSIR-Institute of Genomics and Integrative Biology Campus, New Delhi 110025, India; db@jnu.ac.in; 3 Systems Genomics Laboratory, School of Biotechnology, Jawaharlal Nehru University, New Delhi 110067, India; gauri080191@gmail.com; 4 Federal Research and Clinical Center of Intensive Care Medicine and Rehabilitology, 14-3 Solyanka Street, 109240 Moscow, Russia; avg-2007@yandex.ru; 5 Laboratory of Angiopathology, Institute of General Pathology and Pathophysiology, 125315 Moscow, Russia; daisy29@mail.ru; 6 Institute of Human Morphology, 3 Tsyurupa Street, 117418 Moscow, Russia

**Keywords:** atherosclerosis, inflammation, T cell, B cell, immunity

## Abstract

Atherosclerosis is a well-known global health problem. Despite the high prevalence of the disease, numerous aspects of pathogenesis remain unclear. Subsequently, there are still no cure or adequate preventive measures available. Atherogenesis is now considered a complex interplay between lipid metabolism alterations, oxidative stress, and inflammation. Inflammation in atherogenesis involves cellular elements of both innate (such as macrophages and monocytes) and adaptive immunity (such as B-cells and T-cells), as well as various cytokines cascades. Because inflammation is, in general, a well-investigated therapeutic target, and strategies for controlling inflammation have been successfully used to combat a number of other diseases, inflammation seems to be the preferred target for the treatment of atherosclerosis as well. In this review, we summarized data on targeting the most studied inflammatory molecular targets, CRP, IL-1β, IL-6, IFN-γ, and TNF-α. Studies in animal models have shown the efficacy of anti-inflammatory therapy, while clinical studies revealed the incompetence of existing data, which blocks the development of an effective atheroprotective drug. However, all data on cytokine targeting give evidence that anti-inflammatory therapy can be a part of a complex treatment.

## 1. Inflammation in Atherogenesis

There are three layers of an artery wall: (1) the inner layer, intima, which includes a single layer of endothelial cells, thin basal membrane, and a subendothelial layer of collagen fibers; (2) the media layer, which consists of the smooth muscle cell (SMCs) and a fiber net of collagen and elastin; and (3) the outer layer, the adventitia, which is a loose connective tissue (LCT) [1]. The characteristics of atherosclerosis are atherosclerotic plaque formation in the subendothelial layer, the proliferation of SMC, activated immune cell accumulation, and adventitia thickening in the area of plaque formation. As a rule, various immune cells are present in the arterial wall, but as atherosclerosis progresses, their number elevates significantly [2].

**Figure 1:**
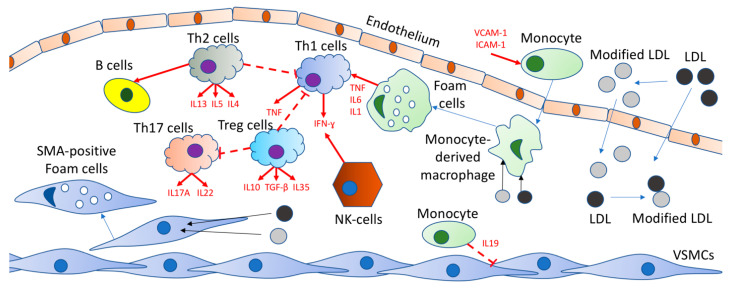
The number of immune cells within the arterial wall is increased at the site of plaque formation. LDL particles undergo multiple modifications and accumulate in the vascular wall where they are internalized by monocyte-derived macrophages and VSMCs, which transform into foam cells. This triggers the productions of cytokines (TNF, IL1, IL6) by foam cells. These cytokines induce the influx and activation of other inflammatory cells and mediate their retention in the plaque, leading to further accumulation of inflammatory cells in the plaque and surrounding adventitia. In particular, these cytokines activate Th1 cells, which produce TNF and IFN-γ, which are also generated by NK cells. Th2 cells also release various cytokines, among which IL3, IL5, and IL4. IL4 mediates the activation of B cells. Th17 cells release IL17A and IL22, while Treg cells generate TGF-β, IL10, and IL35 and block Th17 and Th1 cells.

Normally, immune cells “patrol” the tissue, migrating to the aortic wall and then returning to the blood circulation [3]. In the early stages of atherosclerosis, the expression of adhesion molecules and accelerated migration of monocytes into the aortic wall are noticeable. These signs are generated by high plasma concentrations of low-density lipoproteins (Ldl) and accumulation of liver cholesterol in the aortic wall followed by multiple modifications of LDL (desialylation, oxidation, and others) and high blood pressure, which activates endothelial cells [4]. Monocytes transform into macrophages, which absorb LDL particles and turn into lipid-filled foam cells. Due to the accumulation of modified LDLs caused by macrophages, the cells activate cytokine production. The influx and the activation of other inflammatory cells mediate their retention in the plaque. Inflammatory cells are concentrated in the plaque and surrounding adventitia, as cytokines promote the influx and activation of other inflammatory cells, as well as their retention in the plaque [5]. The immune-cell accumulation within the area of plaque formation, along with the cytokines production and impact, is summarized in Figure 1.

Similar to other tissues, a healthy artery wall has resident macrophages that emerge from the yolk sac during embryonic development [6]. A high level of local ecthyma in both resident and differentiated macrophages from monocytes is a typical atherosclerosis indicator [7].

In addition to monocytes, other myeloid cells mediating inflammatory changes in the aortic wall were also found [8]. The latest research information suggested an idea of the high importance of extracellular neutrophil traps (NETs) consisting of extracellular DNA and neutrophil proteins in the activation of interleukin (IL-1) production and atherosclerosis inflammation [9].

The inflammation of the vessel wall is largely influenced by adaptive immune cells (T-and B-lymphocytes) [10]. Abnormal cells of the T helper (Th) types were found in the aortic wall, and as the disease progressed, the quantity of these cells, their activation state, and the cytokines array produced by them changed. B lymphocytes (B1 and B2) exist in both healthy and atherosclerotic aortas. Along with atherosclerosis development, B1 cells show protective functions by antibody production against various lipids, while B2 cells give rise to disease [11].

A typical sign of late-stage atherosclerosis is so-called unresolved inflammation that is maintained by many factors that involve elevated levels of oxLDLs and high blood pressure [11].

Progressive foam cell storage in plaques is the hallmark of advanced atherosclerosis. The cause of the foam cells’ formation from macrophages is an increased lipid accumulation by the latter. The cells are unable to leave the plaque, so they perish, mainly due to in situ necrosis resulting in necrotic nucleus formation.

The necrotic nucleus is able to cause an imbalance in the tight plaque structure and to lead to its dilaceration, thus causing blood clot formation, which can subsequently lead to the complete blockage of a vessel and cardiovascular complications, such as myocardial infarction and stroke [12].

## 2. Implication of Cytokines in the Atherosclerosis Development

A key role in inflammation is played by cytokines—protein mediators participating in various physiological processes. Cytokines belong to a quite multivarious group of molecules, which includes more than 100 secreted factors. These factors can be classified as (1) interleukins (ILs) (2) tumor necrosis factors (TNFs), (3) interferons (IFNs), (4) transforming growth factors (TGFs), (5) colony-stimulating factors (CSFs), and (6) various chemokines [13].

In response to inflammation and other irritants, T cells, monocytes, macrophages, and platelets, as well as endothelial cells (ECs), SMCs, and adipocytes, produce cytokines. A high level of pro-inflammatory cytokine production is caused by the course of the disease and upregulates the development of atherosclerosis [14]. Cytokine-induced activation of ECs is able to result in endothelial functional disturbance by exasperating the pacing of adhesion molecules and chemokines, which causes immune cells’ (monocytes, neutrophils, and lymphocytes) migration to the atherosclerotic lesion [15]. Cytokines also stimulate migration, proliferation, and growth of SMCs, affecting their functions. At the late stages of atherosclerosis, proinflammatory cytokines lead to disruption of atherosclerotic plaques, various cell apoptosis, and matrix deterioration, which speeds up the destruction of plaques and causes the formation of thrombus [16].

In previous years, cytokines generated by T-helper cells were split up into cytokines generated by type I T-helper cells (Th1) and cytokines generated by type II T-helper cells (Th2). Studies conducted several years ago demonstrate the significant value of type 17 T cells (Th17) and regulatory T cells (Treg) in the original disease development of a large number of different immune disturbances.

Macrophages and T cells significantly affect the activation of Th1-derived cytokines. Cytokines generated by Th2 cells enhance the humoral response [17]. Th17 cells modulate the percolation and activation of myeloid cells in the inflammation focus [18]. Treg cells prevent the activation of all types of T cells and stop immune responses mediated by T cells [19].

## 3. Anti-Inflammatory Treatment of Atherosclerosis

Taken into account, all previously mentioned data on atherogenesis and the role of inflammation in it led to the promising suggestion that anti-inflammatory drugs can be the best treatment option. Different drugs were tested for their efficacy in atherosclerosis and its subsequent cardiovascular complications. Thus, numerous clinical trials were conducted. The data on the most significant in the field are summarized in Table 1.

## 4. IL-1β Targeting

To achieve a biological proactive attitude, the inactive precursor of IL-1β is cleaved by caspase 1. Some proinflammatory cytokines trigger IL-1β production as well as TNF-α [29]. Crystals of cholesterol are able to co-activate NLRP3 inflammasome that controls caspase 1. It is obvious that inflammasome is a tempting target for the abruption of the IL-1β-IL-6 on its way up the flow. During animal experiments, IL-1β showed a proatherogenic effect. For example, in mice, a genetic deficiency of Il-1β decreases atherosclerosis development [30], while in pigs, repeated perivascular administration of recombinant IL-1β speeded up the development of vascular disease by enhancing the generation of pro-inflammatory cytokines and chemokines, including CCL-2, and by stimulating the endothelial expression of adhesion molecules, such as VCAM-1 [31].

To treat gain-of-function diseases (e.g., Muckle–Wells syndrome) treatment, canakinumab, which is a human monoclonal antibody that targets interleukin-1β, was approved. This treatment was practiced for rheumatic inflammatory chronic diseases such as juvenile chronic arthritis or familial Mediterranean fever [32]. In an Ib trial randomized phase I, which involved diabetic patients at high cardiovascular risk, Ridker et al. found that canakinumab treatment did not change cholesterol levels in the plasma but lowered the CRP and IL-6 depending on the drug dosage [32]. Later, the CANTOS (*n* = 10,061) international randomized trial with a follow-up median of 3.7 years showed that despite optimal drug treatment, in the same group, anti-cytokine therapy can reduce major cardiovascular events in people with stable coronary heart disease and highly sensitive CRP >2 mg/L.

Test results performed during the treatment revealed that the participants who, after 3 months, reached an hsCRP concentration of less than 2 mg/L or IL-6 levels below the study median value (1.65 ng/L) showed the greatest result from canakinumab [25]. Additionally, canakinumab treatment reduced oncology incidence and mortality in trial analyses, approximately halved gout attacks, and also worsened heart failure outcomes [33]. These results were achieved due to small but essential infections rising. As the first piece of evidence, CANTOS provided information that, in humans, targeting inflammation is able to lower the frequency of the cardiovascular event regardless of the reduction in lipid levels.

Targeting the NLRP3 inflammasome is an alternative to block the IL-1β, as well as IL-18, pathways. In a low-dose study of colchicine (LoDoCo) simultaneously with secondary prevention of cardiovascular events in 532 patients, colchicine, which prevents the formation of cytoskeletal microtubules and slows down NLRP3 inflammation, was tested [20]. Although the results were promising, there was an increase in the frequency of gastrointestinal colchicine intolerance. Currently, more extensive studies are being conducted in patients with stable coronary heart disease (a low-dose study of colchicine 2 and a study of cardiovascular outcomes of colchicine) in which colchicine is tested. To top it all off, specific low-molecular-weight inhibitors, NLRP3, have been developed to treat various inflammatory processes, such as MCC950. Soon, it is planned to evaluate MCC 950 in patients with atherosclerosis.

## 5. Targeting IL-6

The latest studies have examined a variant of the IL-6 receptor (IL 6R), Asp358Ala, in order to determine the potential of targeting IL-6. This variant assumes that classical IL-6 signaling is able to be disrupted (1) due to a decrease in the number of membrane-bound Il6rs, (2) by increasing the loss of receptors, and (3) by alternative splicing [34]. A meta-analysis was carried out recently to consider this IL-6 receptor polymorphism, which included data collected from more than 2,000,000 patients. Based on the results of analyses of more than 125,000 people without established cardiovascular disease, the researchers were able to determine that the Asp358A genotype was not related to lipid concentration, fasting glucose, systolic blood pressure, or body mass index. At the same time, each inherited copy of Asp358Ala demonstrated an elevation in both the mean IL concentration (14.5%, 10.7–18.4%) and IL6R (34.3%, 30.4–38.2%). There was a reduction in downstream signaling debilitation of hepatic acute-phase reactant displacement, including decreases of CRP by 7.5% (5.9–9.1%) and fibrinogen by 1.0% (0.7–1.3%) [35]. It was found that, in general, patients with the Asp358Ala variant had a reduced risk of developing CHD; however, the risk of developing coronary heart disease lowered with each inherited minor allele by 3.4% [36]. These results demonstrate that the presence of polymorphism in IL6R, regardless of the usual cardiovascular disease risk factors, is caused by a dose-dependent lowering in the accumulation of CRP and fibrinogen and, essentially, lower CHD risk, most likely by reducing the inflammatory response.

### Tocilizumab

Taking into account the benefits that have been observed in patients with Asp358Ala polymorphism, a pharmacological agent that simulates the debilitation of the IL-6 signal could be valuable. Tocilizumab serves as a monoclonal antibody that competitively suppresses IL6R and could be useful in a chronic inflammatory disease such as rheumatoid arthritis [37]. Tocilizumab has been shown to reduce articular inflammation as well as lead to remission of the disease in patients suffering from rheumatoid arthritis [38]. Another study, which was also conducted among patients with rheumatoid arthritis, showed that the endothelium functional impairment improved in comparison to those patients who could not receive a monoclonal antibody. Endothelial dysfunction is defined by disturbance flow-mediated dilatation and aortic stiffness determined by the velocity of the pulse [39]. Comparing the IL 6R variants with tocilizumab revealed a directed, consistent reduction in CRP and fibrinogen [40]. This determines the importance of the capability of IL 6R blockade to provide a new therapy in coronary heart disease treatment.

On the other hand, tocilizumab is able to become a basis for substantial side effects in patients with rheumatoid arthritis. A comparison of two groups, (1) patients with rheumatoid arthritis treated with tocilizumab and (2) patients treated with placebo in a double-blind study, showed that the majority of patients who reported adverse events were from the first group. These adverse events included infections, usually upper respiratory tract infections, and skin side effects. More serious infections appeared on the background of an increased dose of tocilizumab (8 mg/kg). Tocilizumab has also caused various laboratory deviations in patients with rheumatoid arthritis, such as high levels of transaminases and lipids and the absolute number of neutrophils reduction [41].

Hostile changes in the lipid profile due to the possible administration of tocilizumab for atherosclerosis treatment are of the greatest concern. One latest study showed that patients taking tocilizumab had an average elevation in low-density lipoprotein (LDL) levels of 17 mg/dL, and another study found a 22% LDL growth and total cholesterol, as well as a 48% elevation in fasting triglycerides in such patients [42]. These data differ from the data of patients with Asp358Ala polymorphism, in the lipid profile of which non-essential changes were detected. This means that these hostile changes in LDL and high-density lipoproteins may be the result of tocilizumab-specific changes. Such data deprive the drug of the standard of care needed to prescribe a medicine since it can lead to a deterioration of the known cardiovascular risk factor in patients with cardiovascular diseases and those who are simply at risk of these diseases. For this reason, there is a clear need for further research, including randomized trials, to better evaluate the possible efficacy and safety of IL6R blockers in atherosclerotic diseases. Additionally, for the reasons described above, modern IL-6 blockers are not suitable for use in primary or secondary prevention of atherosclerosis. There is a rather urgent need to explore the possibility of further developing therapies targeting IL-6 [43].

## 6. Targeting CRP

Being inhibitors of a key enzyme in the cholesterol biosynthesis pathway, statins are used as a medicine that reduces cholesterol in the human body. On the other hand, statins also lower the level of CRP in humans and transgenic mice with human CRP [44]. Statins reduce the level of CRP regardless of their cholesterol-lowering activity and reduce the level of CRP by suppressing the biosynthesis of CRP by hepatocytes [45]. Nitric oxide also suppresses CRP biosynthesis. Statins are known to produce nitric oxide, so there is a possibility that nitric oxide affects the CRP-reducing effect of statins [46]. Depriving the blood circulation of CRP is not a good idea, because CRP is useful. The treatment that can lower the cholesterol level without lowering the CRP level should be used. This is much preferable to statins, which lower both cholesterol and CRP [45].

The Justification for the Use of Statins in Prevention: An Intervention Trial Evaluating Rosuvastatin (JUPITER) is a large study dedicated to an inalienable role for CRP as a target for therapy in the primary prevention of CV disease. The study involved 17,802 participants (men ≥ 50 years and women ≥ 60 years) with the following characteristics: low cholesterol (LDL < 130 mg/dL and hs-CRP ≥ 2 mg/L) and no history of cardiovascular disease or diabetes. Participants took 20 mg of rosuvastatin or a placebo on a daily basis. The established primary endpoint was the first occurrence of myocardial infarction, stroke, hospitalization for unstable angina, arterial revascularization, or death from cardiovascular disease [27]. This study was terminated prematurely as there was a case rate and mortality growth from CVD in patients treated with rosuvastatin. During the average follow-up period of 1.9 years (max. follow-up period 5 years), rosuvastatin lowered LDL cholesterol by 50% and hs-CRP by 37%, and this result was related to a 44% decrease in the primary trial endpoint (*p* < 0.00001; 95% CI 0.46–0.69). The NTT value, extrapolated over 5 years to avoid one major event, was only 25. This is less than when using statin statins, which are used for primary prevention among patients suffering from more severe hyperlipidemia. Therefore, despite targeting a population beyond the current guidelines and with low LDL cholesterol, JUPITER had a larger value of effect than that of almost all prior statin trials [28].

In the very beginning of 2010, the U.S. Food and Drug Administration (FDA) appealed to JUPITER’s results and decided to expand the rosuvastatin labeling. According to the FDA, rosuvastatin is currently approved as a drug that is able to lower the risk of stroke, myocardial infarction, and revascularization procedures in individuals who, on the one hand, have LDL cholesterol levels within normal limits and no clinically pronounced CHD, but on the other hand, have a high risk depending on age, CRP levels, and the presence of at least one additional risk factor for CVD.

JUPITER also confirmed the “dual-target” hypothesis. The preliminary analysis showed a 65% decrease in major events in patients who achieved LDL cholesterol <70 mg/dL and hs-CRP <2 mg/L versus a 33% decrease in patients who achieved only one or no goals (*p* < 0.0001 in the treatment groups). Thus, JUPITER not only showed that hs-CRP successfully detected a population with a “hidden risk” of cardiovascular disease but also at the same time gave additional confirmations of the potential value of hs-CRP as a therapy target in the primary prevention of cardiovascular disease [47].

## 7. Targeting IFN-γ

IFN-γ is a prototypic Th1 cytokine and appears in atherosclerotic lesions triggered by multiple modified LDL [48]. In the ApoE–/– mouse model, IFN-γ deficiency led to a more stable plaque phenotype. Treatment targeted at IFN-γ with IFNγR gene transfer in ApoE–/– mice resulted in significant 60% atherosclerosis lowering. It is believed that INF-γ is considered to play a proatherogenic role as a suitable target for atherosclerosis treatment [49].

Sortilin is the common receptor of IFN-γ and IL-6. In ApoE–/– mice, the absence of sortilin lowered IFN-γ and IL-6 levels and resulted in a decrease of the atherosclerotic lesion size [50]. Therefore, blocking sortilin is considered to be promising for atherosclerosis treatment strategy. An anti-IFN-γ antibody—Fontolizumab—was examined for the treatment of rheumatoid arthritis or psoriasis. However, the Fontolizumab development was discontinued due to the discrepancy between the results of the clinical trial phase I and the endpoint of the trial [51].

## 8. Targeting TNF-α

Endothelial dysfunction and increased atherosclerosis are revealed in patients with rheumatoid arthritis. TNF-α is a strong pro-inflammatory cytokine that is important for rheumatoid arthritis. TNF-α is also closely related to the mechanisms of speeded up atherosclerosis in rheumatoid arthritis [52].

As a treatment, patients suffering from rheumatoid arthritis received a blocking TNF-α agent, etanercept, and adalimumab. A substantial elevation of total and HDL cholesterol levels was observed [53]. Patients treated with infliximab showed improvements in endothelial function [54]. When targeting inflammatory cytokines, some anti-atherosclerotic drugs are able to slow the inflammatory response according to some clinical trials. Ezetimibe debilitates atherosclerosis caused by lipid-lowering and reduces circulating inflammatory cytokines in the lesion focuses such as monocyte chemoattractant protein (MCP-1) and tumor necrosis factor (TNF-α) [55]. Atorvastatin is also able to defend against moderate atherosclerotic lesions, which decreases cholesterol levels in the blood serum by reducing inflammatory cytokines and also suppresses the macrophages’ storage in the lesion sites [56].

Tanshinone IIA is one of the key diterpenes in Salvia miltiorrhiza. The anti-atherosclerotic effect of tanshinone IIA is related to the suppression of TNF-α-triggered vascular cell adhesion molecule-1 (VCAM-1), intercellular adhesion molecule-1(ICAM-1), and the expression of C-X3-C motif ligand 1 (CX3CL1) [57].

### CANTOS Trial

The trial of canakinumab—CANTOS was a randomized, double-blind, placebo-controlled trial that involved 10.061 cases. All the patients who took part in CANTOS had a myocardial infarction and hsCRP ≥2 mg/L in their history. In the most common and very high-risk group were patients with a “residual risk of inflammation” rather than with a “residual risk of cholesterol”. The study excluded from participation all people with a history of chronic or recurrent infections, oncology other than basal cell carcinoma of the skin, reduced immunity, tuberculosis, or HIV or those who took additional anti-inflammatory drugs. Subcutaneous canakinumab was used every three months in three dosage options: 50, 150, and 300 mg. A combination of non-fatal myocardial infarction, non-fatal stroke, or cardiovascular death (MACE) was a primary endpoint. There was also a second additional MACE endpoint—a hospitalization for unstable angina requiring urgent revascularization (MACE +) [26]. Canakinumab treatment resulted in a notable reduction in both hsCRP and IL-6: the hsCRP median was reduced by 26–41% (depending on the dose) after 48 months (2), and similarly, the IL-6 median was reduced by 19–38% after 12 months. Drug treatment turned out to be ineffective on the LDL-C or HDL-C levels and slightly increased the average level of triglycerides by 4–5% [26].

By the results of the CANTOS study, clinicians have the opportunity to prove that the targeted impact on inflammation has a positive effect on the secondary prevention of cardiovascular diseases; and this benefit depends on the level of cholesterol. This is a reminder of the existence of new treatment options for those who have a residual risk of inflammation, which, despite an appropriate reduction in LDL-C, is determined by a consistently high hsCRP level [58]. Despite the successful elimination of traditional risk factors for CVD, neither clinicians nor patients should believe that there are no more ways to reduce the risks. Despite vital advances in pharmacological and life-saving interventions (primary and secondary prevention), CVD prevails over other causes of death, and therefore the issue is still quite relevant.

In addition to CANTOS, other anti-inflammatory drugs, including low-dose methotrexate: colchicine in the LoDoCo2 and COLCOT studies, as well as in studies involving other modulators of IL-1, IL-6, and NLRP3 inflammation, are currently being additionally tested as part of a study to reduce cardiovascular inflammation [59].

Even though most of the CANTOS patients received lipid-lowering therapy with low baseline LDL cholesterol levels, it remains to be investigated whether anti-inflammatory therapy will have a positive effect on patients who receive PCSK9 inhibitors with even lower LDL-C levels [60].

## 9. Conclusions

Inflammation plays a very important role in the development of atherosclerosis as one of its triggers. Cytokines accompany atherogenesis at all its stages, which only emphasizes the importance of the role of inflammation in the pathogenesis of atherosclerosis.

Of course, the choice of cytokines as a therapeutic target looks very logical and promising. Indeed, studies on animal models support the hypothesis of the atheroprotective effect of anti-inflammatory drugs. However, according to the results of human studies and clinical trials, the current level of knowledge about the pathogenesis of atherosclerosis is insufficient to develop a truly effective treatment for this disease. Nevertheless, the use of anti-inflammatory drugs can become part of the complex therapy of atherosclerosis, aimed simultaneously at several aspects of atherogenesis.

## Figures and Tables

**Table 1:** Summary of clinical trials data, which examined the effects of anti-inflammatory drugs on various cardiovascular conditions. The table contains the name of the trial, the name of the tested drug, the condition in which it was examined, the effects that were observed, and the study reference.

Trial Name	Tested Drug	Condition	Effects	Reference
LoDoCo	Colchicine (0.5 mg/d)	stable coronary artery disease	Prevention of recurrent cardiovascular events	[20]
LoDoCo2	Colchicine (0.5 mg/d)	stable coronary artery disease	efficacy and safety of low-dose colchicine for secondary prevention	[21]
COLCOT	colchicine (0.5 mg/d) to standard-of-care therapy	patients after MI	Prevents cardiac events	[22]
CIRT	low-dose weekly methotrexate (10–25 mg)	coronary artery disease	Reduction of plasma biomarkers of inflammation	[23]
ENTRACTE	Tocilizumab and Etanercept	moderate-to-severe rheumatoid arthritis	Risk of occurrence of the MACE of 1.43 or higher was observed	[24]
CANTOS	Canakinumab	participants with prior MI and elevated CRP levels	Lowering of the frequency of the cardiovascular events regardless of the reduction in lipid levels	[25,26]
JUPITER	20 mg of rosuvastatin per day	low cholesterol (LDL < 130 mg/dL and hs-CRP ≥ 2 mg/L) and no history of cardiovascular disease or diabetes	Terminated prematurely as there was a case rate and mortality growth from CVD	[27,28]
